# Simultaneous identification of clinically relevant single nucleotide variants, copy number alterations and gene fusions in solid tumors by targeted next-generation sequencing

**DOI:** 10.18632/oncotarget.25229

**Published:** 2018-04-27

**Authors:** Duarte Mendes Oliveira, Teresa Mirante, Chiara Mignogna, Marianna Scrima, Simona Migliozzi, Gaetano Rocco, Renato Franco, Francesco Corcione, Giuseppe Viglietto, Donatella Malanga, Antonia Rizzuto

**Affiliations:** ^1^ Department of Experimental and Clinical Medicine, University “Magna Graecia”, Catanzaro, Italy; ^2^ Department of Health Sciences, University “Magna Graecia”, Catanzaro, Italy; ^3^ Pathology Unit, Istituto Nazionale Tumori “Fondazione G. Pascale”, IRCCS, Naples, Italy; ^4^ Pathology Unit, Second University of Naples, Italy; ^5^ Department of Medical and Surgical Sciences, University “Magna Graecia”, Catanzaro, Italy; ^6^ Interdepartmental Center of Services (CIS), University “Magna Graecia”, Catanzaro, Italy; ^7^ UOC Chirurgia Generale, Azienda Ospedaliera dei Colli, Napoli, Italy; ^8^ Biogem scarl, Institute of Genetic Research, Ariano Irpino (AV), Italy

**Keywords:** solid tumors, oncomine focus assay, copy number variation, gene fusions

## Abstract

In this study, we have set-up a routine pipeline to evaluate the clinical application of Oncomine™ Focus Assay, a panel that allows the simultaneous detection of single nucleotide hotspot mutations in 35 genes, copy number alterations (CNAs) in 19 genes and gene fusions involving 23 genes in cancer samples. For this study we retrospectively selected 106 patients that were submitted to surgical resection for lung, gastric, colon or rectal cancer.

We found that 56 patients out of 106 showed at least one alteration (53%), with 47 patients carrying at least one relevant nucleotide variant, 10 patients carrying at least one CNA and 3 patients carrying one gene fusion. On the basis of the mutational profiles obtained, we have identified 22 patients (20.7%) that were potentially eligible for targeted therapy.

The most frequently mutated genes across all tumor types included KRAS (30 patients), PIK3CA (16 patients), BRAF (6 patients), EGFR (5 patients), NRAS (4 patients) and ERBB2 (3 patients) whereas CCND1, ERBB2, EGFR and MYC were the genes most frequently subjected to copy number gain. Finally, gene fusions were identified only in lung cancer patients and involved MET [MET(13)–MET(15) fusion] and FGFR3 [FGFR3(chr 17)–TACC3(chr 11)].

In conclusion, we demonstrate that the analysis with a multi-biomarker panel of cancer patients after surgery, may present several potential advantages in clinical daily practice, including the simultaneous detection of different potentially druggable alterations, reasonable costs, short time of testing and automated interpretation of results.

## INTRODUCTION

Precision Medicine is a term that is used to describe how genetic information about patients is used to diagnose and treat their disease [1, 2]. In oncology, the success of precision medicine relies on validated biomarkers that allow accurate prognosis and prediction of response to therapy [3].

Until recently, the use of genetic biomarkers to direct therapy of oncological patients has been hampered by the necessity to analyse one biomarker at a time [4, 5]. For example, predicting the response to trastuzumab in breast cancer patients is dictated by positivity to HER2 [6–8], sensitivity to the anti-EGFR inhibitor gefitinib in NSCLC patients is predicted by EGFR mutations [9, 10] and resistance to the anti-EGFR monoclonal antibody cetuximab in advanced colorectal cancer patients is predicted by KRAS mutations [11].

The introduction of Next Generation Sequencing (NGS) techniques has allowed the development of assays for the simultaneous analysis of multiple biomarkers [12]. Whole genome and/or exome NGS studies have started to depict the genetic landscapes of a number of human cancer types [13, 14]. However, due to the huge amount of data generated by these type of studies, the identification of clinically relevant alterations in specific patients is often doubtful, which has delayed the introduction of NGS in the clinical setting [15]. In addition, NGS is expensive, time consuming and presents important technical challenges including source, quantity and quality of the DNA to be sequenced [16].

The development of NGS panels containing a relatively small number of cancer-associated genes has paved the way to the simultaneous detection of multiple genetic alterations in tumors, soon after surgical resection, allowing rapid therapeutic decision [17–19]. Amplicon-based NGS offers a sensitive, cost-effective approach for detecting multiple genetic alterations with a minimum amount of DNA [20, 21] that can be performed on DNA extracted from formalin-fixed paraffin-embedded (FFPE) tissues [22–24]. These panels include, among others, the Ion AmpliSeq™ Cancer Hotspot Panel v2 and the Ion AmpliSeq Colon and Lung Cancer Panel. The first panel is used to identify mutations in genomic “hot spot” regions that are frequently mutated in human cancer genes. It is designed to cover 2,800 COSMIC mutations from 50 oncogenes and tumor suppressor genes. The second panel is a multiplex PCR-based method that encompasses 1825 mutational hotspots in 22 genes related to colon and lung cancer [25–27]. Although these panels have been successfully used in different validation studies to identify mutations in KRAS, NRAS and BRAF genes by NGS [27, 28] and have been proposed to be used as routine assays to genotype cancer patients [25], they present some limitations, the most important of which is the fact that they detect only SNVs and/or small indels.

Recently, a panel denoted Oncomine™ Comprehensive Panel (OCP) [29] as well as the smaller panel denoted Oncomine™ Focus Assay (OFA) were developed. OFA is a multi-biomarker panel that enables the simultaneous detection of single nucleotide variants (SNVs), indels, CNAs and gene fusions in a single workflow across 52 genes that are relevant to solid tumors and druggable [30, 31]. OFA and OCP present several advantages over other conventional panels, including the necessity of low amount of nucleic acids and the possibility to use either fresh or FFPE samples.

In this study, we have set up a simple, routinary pipeline from surgery to the laboratory to evaluate the clinical application of OFA with the aim of improve the current characterization of surgically treated cancer patients. This pipeline comprises: i) retrospective selection of patients subjected to surgical resection for lung (LC), gastric (GC), colon (CC) or rectal (RC) cancer, ii) retrievement of FFPE specimens and dissection of the tumor area, iii) molecular analysis of DNA and RNA extracted from dissected tumors using OFA, iv) selection of druggable target(s) for each patient and identification of the corresponding targeted therapy.

## RESULTS

Using the Oncomine™ Focus Assay, we have retrospectively analyzed 4 different cohorts of FFPE tissue specimens surgically resected from patients affected by LC, GC, CC and/or RC. Patients accrued for this study were 106 (106 primary tumors: 28 LC, 22 GC, 31 CC, 25 RC; 6 metastatic lymph nodes). DNA could be properly analyzed in all cases whereas RNA alterations were evaluable in 96 samples. It is of note that the 6/10 samples that could not be analyzed at the RNA level were GC, which represented the oldest samples in the cohort under analysis, with some patients dating back to 2004. Samples that weren't evaluable (N.E.) in the RNA workflow were indicated in Tables 1-4. See Supplementary Tables 1-4 for clinical-pathological features of the patients under study. Supplementary Figure 1 describes the workflow and representative pictures of the output of OFA analysis.

**Table 1:** Alterations detected in NSCLC patients (n=28)

Patient	SNV	Allele Frequency	Validation (Sequencing)	CNA	Validation (RT-PCR)	Fusion
LC1						met(13)-met(15)
LC2						
LC3						
LC4						
LC5						
LC6						
LC7				CCND1 (CN=8.5)	Yes	
LC8	PIK3CA-G546K	8%	Sanger	FGFR1 (CN=5.5)	Yes	
LC9						
LC10						
LC11						
LC12	BRAF-V600E	37%	Sanger			N.E.
LC13						
LC14	PIK3CA-C420R	26%		ERBB2 (CN=8)	Yes	N.E.
LC15	NRAS-G61K	40%				
LC16						
LC17						
LC18	KRAS-G12D	58%	Sanger			
LC19						fgfr3 (17)- tacc3(11)
LC20	KRAS-G12C	13.5%	Sanger			
LC21						N.E.
LC22						
LC23	KRAS-G12D		Sanger			
LC24	EGFR-L858R	25%				
LC25	EGFR-GLU746_ALA750DEL	20%				
LC26						met(13)-met(15)
LC27						
LC28						

N.E. – Non Evaluable.

**Table 2:** Alterations detected in Gastric cancer (GC) patients (n=22)

Patient	SNV	Allele Frequency	Validation (Sequencing)	CNA	Validation (RT-PCR)	Fusion
G1	ND					
G2	ND			ERRB2 (CN=5.29) MYC (CN=8.5)	Yes	
G3	ND					
G4	ND			CDK6 (CN=56.13) ERBB2 (CN=39.92) CCND1 (CN=7)	Yes	
G5	KRAS-G12V	21%	Sanger			N.E.
G6	ND			CCND1 (CN=4.88)	Yes	
G7	ND					
G8	ND					N.E.
G9	KRAS-G12D	29%	Sanger			
G10	PIK3CA-E546R MAP2K1-L57T	8% 5%	Sanger ^*^			
G11	PIK3CA-E545L	6%				
G12	ND					
G13	PIK3CA-M1043I ERBB2-R896C JAK3-R657G	18% 12% 5%	^*^	CCND1 (CN=6.5) MYC (CN=6.5)	Yes	
G14	ND					N.E.
G15	ND			ERBB2 (CN=10.62)	Yes	
G16	ND					
G17	ND					N.E.
G18	ND					
G19	EGFR-A289D ERBB2-R896C	9% 18%				
G20	PIK3CA-H1047R PIK3CA-H1048Y JAK3-R657G	24% 58% 4%	^*^			
G21	PIK3CA-E547K KIT-D816N SMO-R290H ERBB3-E332K	62% 2% 100% 80%	Sanger ^*^ Sanger			N.E.
G22	NRAS-A146T NRAS-G61L KRAS-G13S	5% 42% 13%	Sanger			N.E.

N.E. – Non Evaluable.

^*^ SNV not confirmed Sanger sequencing.

**Table 3:** Alterations detected in Colon cancer (CC) patients (n=31)

Patient	SNV	Allele Frequency	Validation (Sequencing)	CNA	Validation (RT-PCR)	Fusion
CC1	BRAF-V600E	32%	Sanger			
CC2	MTOR-T1977K KRAS-G12D	7,5% 16%				
CC3						
CC4	KRAS-A146T	8%		FGFR1 (5)	Yes	
CC5	KRAS-G12C	14%				
CC6	PIK3CA-E542K KRAS-G12V	30% 49%	Sanger Sanger			
CC7	PIK3CA-H1047R BRAF-V600E	23% 22.5%	Sanger			
CC8	PIK3CA-G1049C BRAF-V600E	20% 19%	Sanger Sanger			
CC9						
CC10						
CC11	KRAS-A146T	44.5%				
CC12	NRAS-G61K	25%				
CC13			NGS Panel^**^			
CC14						
CC15						
CC16	PIK3CA-H1047Y BRAF-V600E	16.5% 9%				
CC17	KRAS-G12S PIK3CA-E542K	37.5% 39%				
CC18	KRAS-G12V	37.5%				
CC19	KRAS-A146T	57%				
CC20						
CC21			NGS Panel			
CC22						
CC23						
CC24	KRAS-G12V PIK3CA-E545K	31% 39%				
CC25	KRAS-G13D	15%				
CC26	KRAS-G12A ERBB3-G332K	15% 5.5%				
CC27						
CC28	KRAS-G12S EGFR-G719D NRAS-G61K	33% 10% 38%	Sanger			
CC29	KRAS-G13D PIK3CA-E545K	31% 36%				
CC30	RET-A883V KRAS-G12D AKT1-E17K	9% 37% 11%				
CC31	KRAS-G13D KRAS-A146T	10% 38%				

^**^ The NGS Panel used is the Comprehensive Cancer Panel from Thermofisher.

**Table 4:** Alterations detected in Rectal cancer (RC) patients (n=25)

Patient	SNV	Allele Frequency	Validation (Sequencing)	CNA	Validation (RT-PCR)	Fusion
RC1	ND		NGS Panel^**^			
RC2	ND		NGS Panel^**^	FGFR1 (7.5)	Yes	
RC3	ND					
RC4	ND					
RC5	ND					
RC6	BRAF-V600E	10%	Sanger			
RC7	KRAS-A146P	16%				
RC8	KRAS-G12V	41%	Sanger			
RC9	ND					
RC10	ND					
RC11	ND					
RC12	ND					
RC13	KRAS-A146T ERBB2-S310F	47% 40%				
RC14	KRAS-G13D	15.5%	Sanger			
RC15	ND					
RC16	ND					
RC17	ND					
RC18	KRAS-G12V PIK3CA-E545K EGFR-A289D	27% 19% 6%	Sanger Sanger ^*^			
RC19	KRAS-G13D AKT1-E17K	23% 13%				
RC2	ND					
RC21	ND					
RC22	KRAS-G12V	38%				
RC23	ND					
RC24	KRAS-G12S PIK3CA-E545K	24% 12%	Sanger Sanger			
RC25	ND					

^*^ SNV not confirmed by Sanger sequencing.

^**^ The NGS Panel used is the Comprehensive Cancer Panel from Thermofisher.

DNA and RNA were extracted from primary tumors (n=106) and metastatic lymph nodes (n=6) and quantified as described in Materials and Methods. Once checked for quality, we performed NGS analysis on the Ion Torrent platform with OFA. Results obtained for LC, GC, CC and RC are summarised in Tables 1-4, respectively. Data for the lymph node metastasis are summarized in Table 5.

**Table 5:** Alterations detected in metastatic lymph nodes (n=6) and corresponding primary tumors

Patient	SNV (Primary Tumor)	CNA (Primary Tumor)	SNV (Metastasis)	Allele Frequency	CNA (Metastasis)	Validation (RT-PCR)
G4-M		CDK6 (CN=56) ERBB2 (CN=40) CCND1 (CN=7)			CDK6 (CN=65) ERBB2 (CN=50) CCND1 (CN=8)	Yes Yes Yes
G13- M	PIK3CA-M1043I ERBB2-R896C JAK3-R657G	CCND1 (CN=5)	PIK3CA-M1043I ERBB2-R896C JAK3-R657G	33% 14% 6%	CCND1 (CN=6.5)	Yes
G20-M	PIK3CA-H1047R PIK3CA-H1048Y JAK3-R657G		PIK3CA-H1047R PIK3CA-H1048Y MET-Y1253C JAK3-R657G	24% 38% 5% 6%		
CC28-M	KRAS-G12S EGFR-G719D NRAS-G61K		KRAS-G12S NRAS-G61K RAF1-T421I	32% 54% 8%		
CC30-M	RET-A883V KRAS-G12D AKT1-E17K		KRAS-G12D RET-A883V AKT1-E17K	30% 9.5% 11%		
CC31-M	KRAS-G13D KRAS-A146T		PIK3CA-E365K KRAS-G13D KRAS-A146T	17% 62.5% 1%		

**Figure 1:**
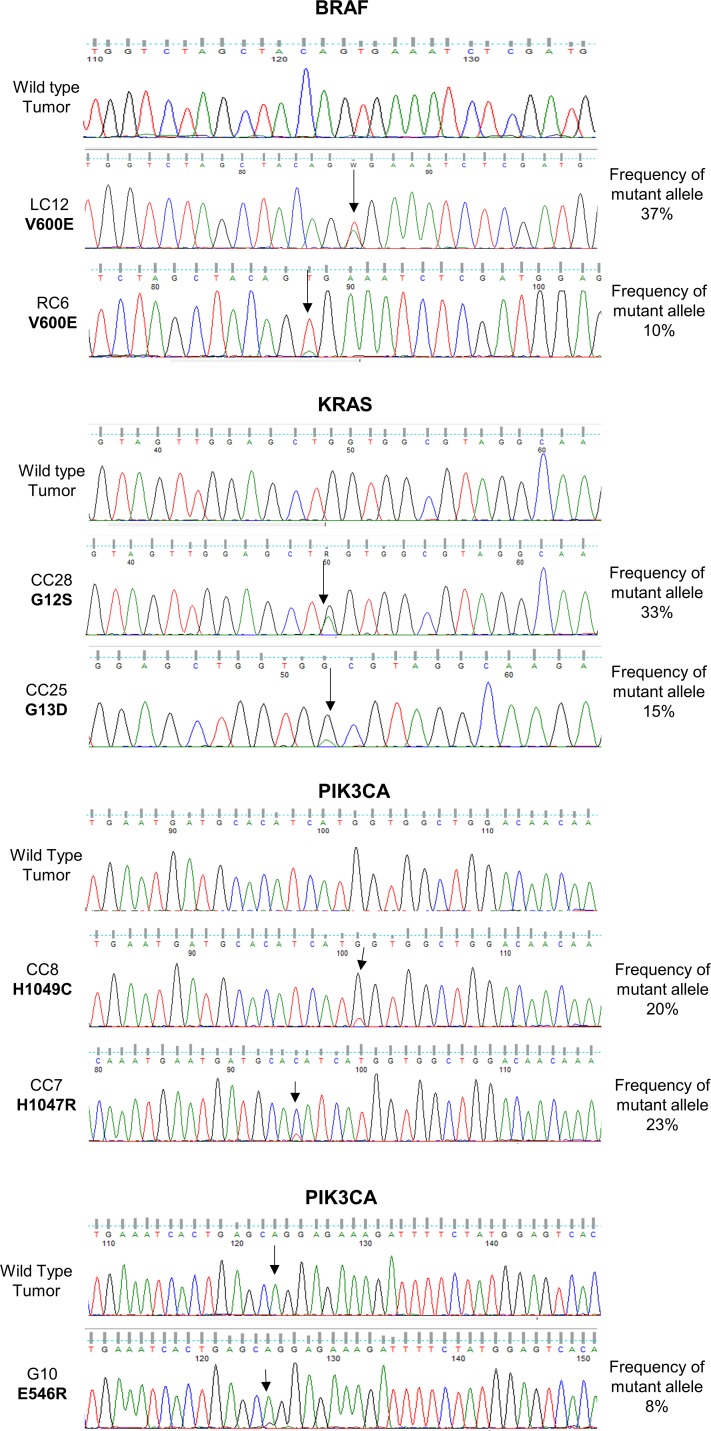
Validation of SNVs identified by Oncomine™ Focus Assay Traditional Sanger DNA sequencing of SNVs detected by the Oncomine™ Focus Assay in BRAF, KRAS and PIK3CA genes.

Sanger DNA sequencing was used to confirm SNVs, with different enrichment of the mutant allele. Sanger DNA sequencing was performed for 20 patients. See Figure 1 for validation of KRAS (patients CC28, CC25), BRAF (patients LC12, RC6) and PIK3CA (patients CC8, CC7 and G10) mutations. Mutations detected in SMO (patient G21), MAP2K1 (patient G10) and MET (patient G20-M) were also validated (data not shown). Expectedly, we found that the Sanger DNA sequencing technique was much less sensitive in detecting SNVs than OFA. In fact as shown in the electropherograms of Figure 1 the height of the peaks detected by DNA sequencing was directly proportional to the frequency of the alleles. Moreover, in agreement with several previous studies, the lower limit of allele frequency that allowed detection by Sanger DNA sequencing was about 8%.

**Figure 2:**
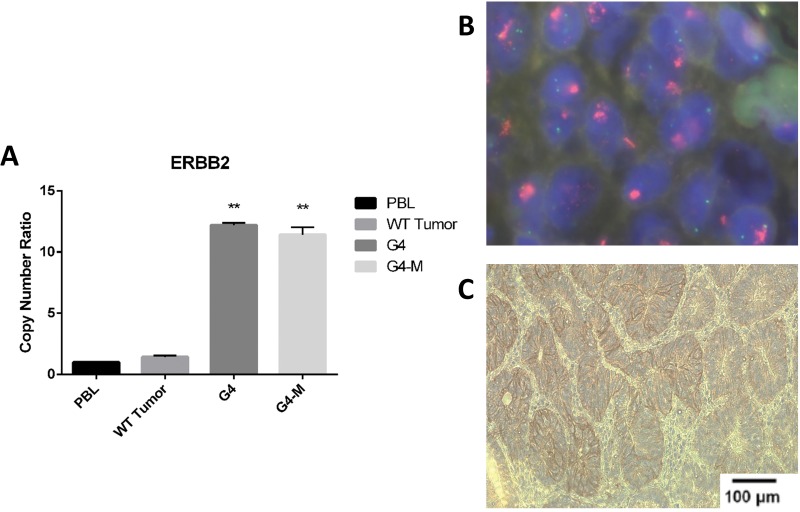
Validation of gene amplification identified by Oncomine™ Focus Assay **(A)** Quantitative real-time PCR in patients (G4 and G4-M) with amplified ERBB2 gene. PBL, peripheral blood lymphocytes; tumors showing no ERBB2 amplification, statistical significance indicated by number of stars in each patient when confronted with PBL. **(B)** FISH analysis of ERBB2 in a patient (G4) with amplified ERBB2 gene. **(C)** Immunostaining analysis of amplified ERBB2 protein in patient G4.

CNAs were confirmed by qPCR. See Figure 2A for validation of CNAs in ERBB2 in GC patient G4 by qPCR and Figure 2B by FISH analysis. See Supplementary Figure 2 for validation of all remaining CNAs (CDK6, CCND1, MYC and FGFR1) by real-time PCR. Immunostaining was also used to determine whether the detected gene amplification was mirrored into increased protein expression. In Figure 2C we report a representative example of ERBB2 protein expression.

Overall, in OFA DNA analysis reported here, we found that 53 of the 106 collected samples showed at least one alteration (50%). The remaining samples showed no readily identifiable alteration (53/106, 50%). Of the 53 samples that presented positive calls in OFA DNA analysis, 26 presented 1 alteration, 19 presented 2 alterations, 6 presented 3 different alterations and 2 samples presented more than 3 alterations. The most frequently mutated genes across all tumor types included KRAS (30 patients), PIK3CA (16 patients), BRAF (6 patients), EGFR (5 patients), NRAS (4 patients), and ERBB2 (3 patients). Analysis of CN data revealed that the most frequent copy number gain occurred in the genes encoding CCND1 and ERBB2 (4 patients, 4%), followed FGFR (3 patients, 2.8%) and MYC (2 patients, 1.9%). We identified CNAs in a total of 10 patients. In some cases (n=4/10), CN gains co-occurred with other relevant hotspot mutations, whereas in the remaining six cases CN gains revealed isolated amplifications and no additional hotspot mutations.

In the OFA RNA analysis 3 of the 106 samples presented positive calls. A gene fusion involving MET [denoted MET(13)–MET(15) fusion] was identified in two LC patients (7.1%) and a gene fusion involving FGFR3 (FGFR3-TACC3) was identified in one LC patient (3.6%). No gene fusion was identified in GC, CC or RC. Tumors harbouring gene fusions presented no other alteration, indicating that the identified fusions could drive tumorigenesis in the affected patients.

### OFA analysis in LC patients

**Figure 3:**
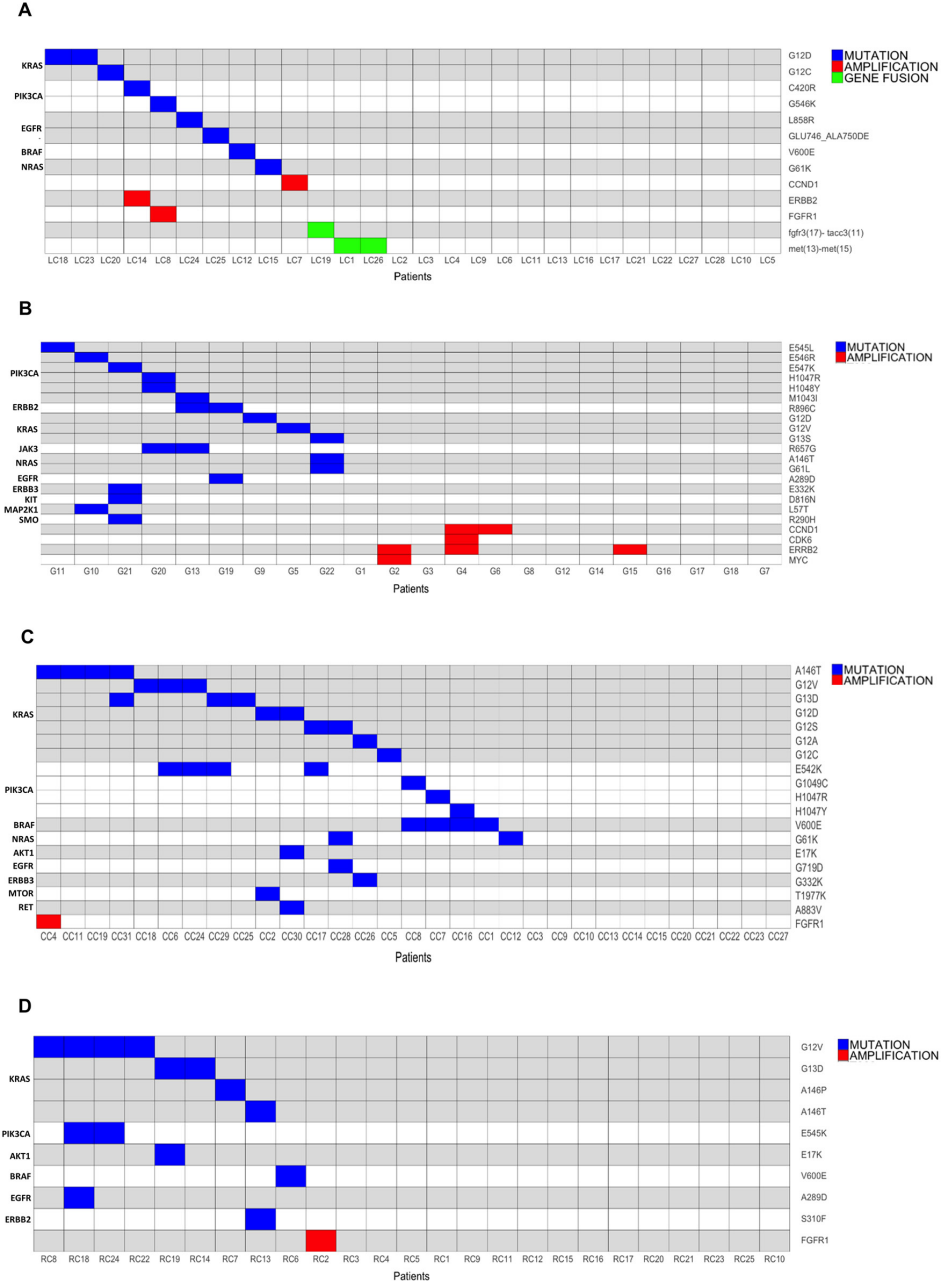
Mutation, amplification and gene fusion profiles of the tumors under analysis **(A)** Graphical representation of the alterations identified in LC patients. Horizontal lines indicate SNVs or CNAs; vertical lines indicate patients. **(B)** Graphical representation of the alterations identified in GC patients. Horizontal lines indicate SNVs or CNAs; vertical lines indicate patients. **(C)** Graphical representation of the alterations identified in CC patients. horizontal lines indicate SNVs or CNAs; vertical lines indicate patients. **(D)** Graphical representation of the alterations identified in RC patients. horizontal lines indicate SNVs or CNAs; vertical lines indicate patients.

**Figure 4:**
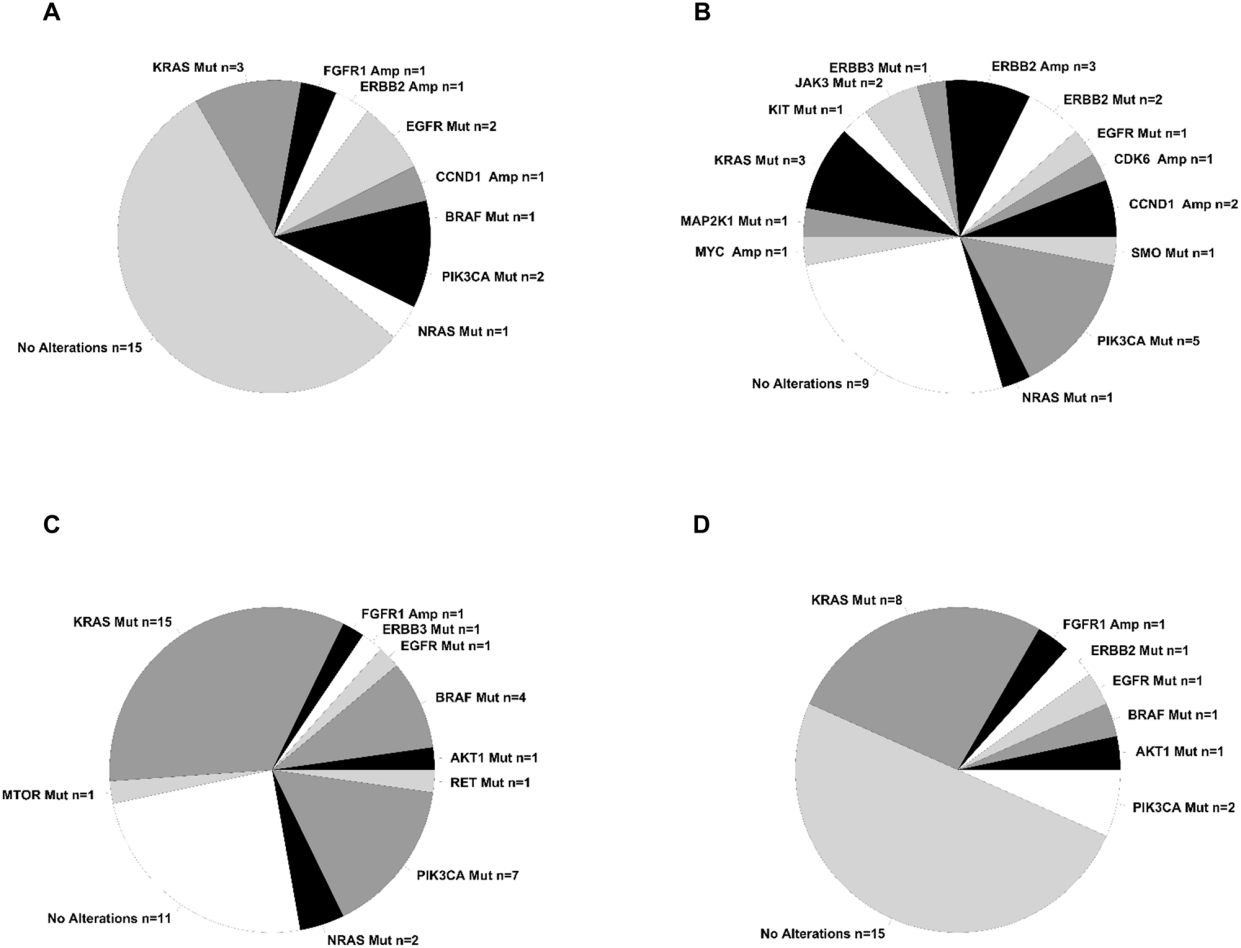
Summary of alterations identified in the tumors under analysis Pie chart containing absolute numbers of each specific alteration identified. **(A)** Summary of the alterations identified in LC patients. **(B)** Summary of the alterations identified in GC patients. **(C)** Summary of the alterations identified in CC patients. **(D)** Summary of the alterations identified in RC patients.

Overall we identified 13 different potentially druggable molecular alterations (7 different SNVs, 1 small indel, 3 CNAs and 2 gene fusions) in 13/28 LC patients (47%). Patients that presented gene fusions were negative for CNAs and/or SNVs. Conversely, patients that presented CNAs (FGFR1 in LC8, ERBB2 in LC14) also showed the presence of PIK3CA mutations. See Figure 3A for a schematic representation of the results of OFA analysis in LC and Figure 4A for a summary of the alterations identified.

**Figure 5:**
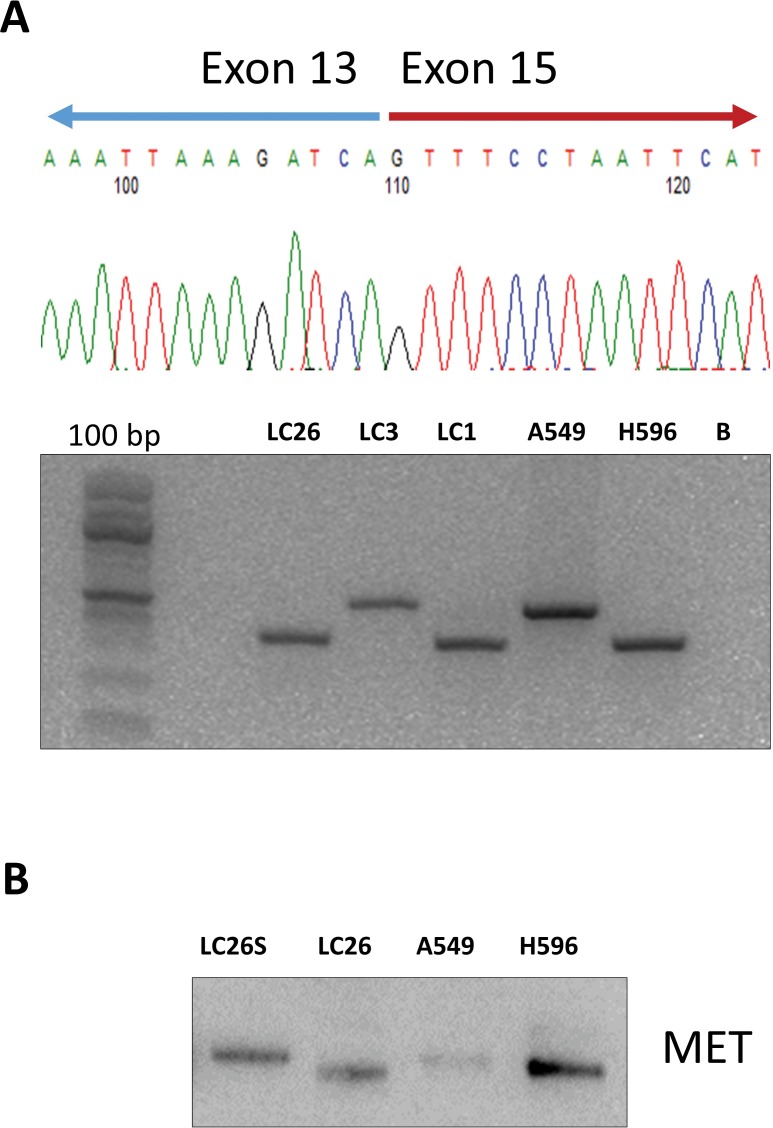
Immunoblot analysis of MET and phospho-MET in NSCLC **(A)** NSCLC samples positive (LC1, LC26) and negative (LC23) for the MET(13)–MET(15) fusion were subjected to semi-quantitative RT-PCR performed on RNA extracted from tumors. The cell line H596 was used as positive control and Chromatogram from Sanger sequencing of the breakpoint of the MET(13)–MET(15) fusion in patient LC26. **(B)** NSCLC sample positive (LC26) and negative (LC25 with normal counterpart) for the MET(13)–MET(15) fusion were subjected to immunoblot with anti-MET. Protein lysate from A549 cells was used as negative control. Protein lysate from NCI-H596 cells was used as positive control for the MET(13)–MET(15) fusion.

Oncogenes activated by somatic SNVs were 5: KRAS (G12D, G12C) in 3 patients (11%), PIK3CA (G546K, C420R) in 2 patients (7%), NRAS (G61K) in 1 patient (3.5%), BRAF (V600E) in 1 patient (3.5%) and EGFR in 1 sample (L858R). EGFR also presented a small deletion (GLU746_ALA750DEL). CNAs were detected in 3 samples: 1 patient presenting FGFR1 amplification (CN= 5.5), 1 patient presenting CCND1 amplification (CN= 8.5) and 1 patient presenting ERBB2 amplification (CN= 8). In addition, we found 3 patients that presented gene fusions involving the genes MET (n = 2) and FGFR3 (n =1), respectively. Patients LC1 and LC26 presented an intragenic fusion in the MET gene [MET(13)–MET(15) fusion] that was caused by skipping of MET exon 14, due to an aberrant splicing event that led to link exon 13 to exon 15. Notably, in the Ion Reporter output, the two LCs that were reported positive for MET (13)–MET(15) fusion presented a high number of reads (>15,000) specific for the fusion. The presence of MET(13)–MET(15) fusion in patients LC1 and LC26 was validated by performing RT-PCR followed by Sanger sequencing to identify the breakpoints (Figure 5A).

The MET(13)–MET(15) fusion has been reported to occur at low frequency in LC patients [32], leading to a shorter MET protein lacking exon 14 (47 aminoacids) that present increased stability and activity [33]. Accordingly, immunoblot analysis of patient LC26 demonstrated the presence, in cancer tissue, of an abnormal MET protein with lower molecular weight that was expressed at increased levels, compared with the MET protein expressed in the corresponding normal tissue of the same patients (Figure 5B).

**Figure 6:**
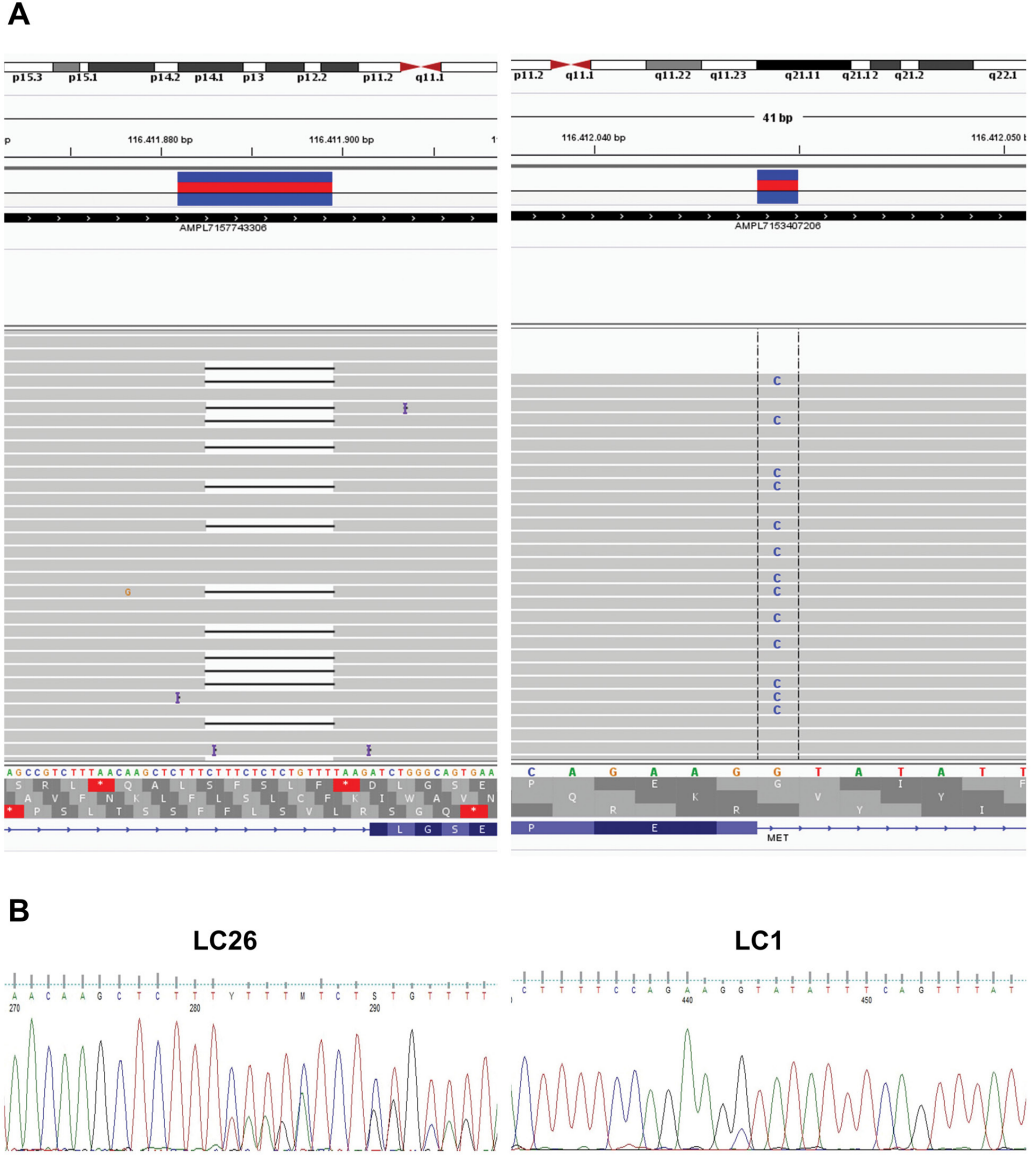
Genomic alterations that promote MET exon 14 skipping in NSCLC **(A)** NGS analysis of the mutations detected in intron 14 in patients LC26 and LC1 (left and right panels, respectively). **(B)** Chromatograms from Sanger sequencing of the genomic mutations in intron 14 of patients LC26 and LC1.

To identify the molecular alteration(s) on DNA that were responsible for the skipping of exon 14 in LC samples reported positive for MET(13)–MET(15) fusion, we designed a custom NGS panel that covered 1578 base pairs of the MET genomic region, spanning intron 14, exon 14 and intron 15 of the MET gene. By sequencing the genomic DNA of the two patients positive for the MET(13)–MET(15) fusion, we found a heterozygous mutation in the acceptor site of exon 14 in patient LC1 and a small deletion of 14 bp in intron 13 in patient LC26 that abolished the donor site of exon 13. DNA alterations in patients LC1 and LC26 were confirmed by Sanger sequencing and manually visualized on the integrative genomics viewer (IGV) [34]. See Figure 6A and 6B.

In addition, patient LC19 presented a chromosomal translocation involving the gene encoding FGFR3 on chromosome 4 and the gene encoding TACC3 on the same chromosome [denoted FGFR3(chr 17) – TACC3(chr 11) fusion] presenting 23 reads specific for this fusion transcript. Subsequently, we performed RT-PCR to confirm the presence of the FGFR3(chr 17) – TACC3(chr 11) fusion in the mRNA of LC19. However, despite several attempts we were not able to validate this fusion by RT-PCR. See Supplementary Figure 3 for a representative experiment. Notably, no FGFR3(chr 17) – TACC3(chr 11) fusion was identified in the analysis performed by RT-PCR on RNA extracted from 69 LC samples that include the 22 samples under study here. These results suggest that the FGFR3-TACC3 fusion identified in patient LC19 may be a false positive.

No fusion involving the ALK or the RET genes were detected in the LC analysed, confirming the relative low frequency of these alterations.

### OFA analysis in GC patients

In the case of GC we identified at least one alteration in 13 patients (63%) distributed across 13 different genes. Of the different alterations identified, 18 were SNVs and 4 were CNAs. No gene fusion was detected. See Figures 3B and 4B, respectively for the results of OFA analysis in GC. Three patients showed single SNVs, 3 patients showed the simultaneous presence of 2 SNVs (in EGFR and ERBB2, in PIK3CA and MAP2k1, in KRAS and NRAS), and 4 patient showed the simultaneous presence of 4 SNVs.

Multiple somatic SNVs were found in PIK3CA (E545L, E546R, E547K, M1043I, H1047R, H1048Y) (7 patients, 23%), in JAK3 (R657G) and in KRAS (G12D, G12V) 2 patients (6.5%). SNVs in MAP2K1 (L57T) and ERBB2 (R896C) were observed in one patient (3.3%). Finally, one patient (3.3%) showed the simultaneous presence of SNVs in EGFR (A289D) and ERBB2 (R896C) whereas another patient showed the simultaneous presence of SNVs in KIT (D816N), ERBB3 (E322K), and SMO (R890H) genes.

CNAs were detected in the gene encoding ERBB2 (CN range = 5-40) and CCND1 (CN range = 5-7) in three patients, in the gene encoding MYC (CN range = 6-8) in two patients and in the gene encoding CDK6 (CN range = 55) in one patient. Two patients showed a single CNA, two patients showed the simultaneous presence of two (MYC and ERBB2, CCND1 and MYC, respectively) or three (ERBB2, CCND1 and Cdk6) CNAs. None of the gene fusions present in the panel was detected in GC.

### OFA analysis in CC patients

By analysis of 31 CC patients we identified 19 different molecular alterations in 10 different genes in 20/31 (64.5%) of the patients analyzed. Of these, 18 were SNVs and 1 was CNA (3%). See Figures 3C and 4C for a summary of the results obtained in the OFA analysis in CC.

Overall we found somatic SNVs in KRAS (G12D, A146T, G12V, G12C, G12S, G13D) in 15 patients (48.4%), in PIK3CA (E542K, E545K, G1049C, H1047R, H1047Y) in 7 patients (22.6%), in BRAF (V600E) in 4 patients (12.9%), NRAS (G61K) in 2 patients (6.5%) and MTOR (T1977K), AKT1 (E17K) and RET (A873V) in one patient (3.2%).

CNAs were detected only in 1 CC (FGFR1, CN = 5). The amplification of FGFR1 occurred in a sample with KRAS mutation. Conversely, eight CC patients showed the presence of single SNVs (KRAS, NRAS, BRAF) and seven patients showed the simultaneous presence of multiple SNVs. Whereas mutations in BRAF, KRAS and NRAS were mutually exclusive, several patients showed the simultaneous presence of mutations in PIK3CA and KRAS or BRAF. One CC patient showed the simultaneous presence of mutations in MTOR and KRAS.

### OFA analysis in RC patients

Overall we identified 10 different molecular alterations in a total of 10 out of 25 RC patients (40%). Of these, 9 were different SNVs and one was a CNA (4%).

SNVs were detected in KRAS (G12V, A146P, A146T, G13D, G12S) in 8 patients (32%), PIK3CA (E545K, E542K) in two patients (8%), ERBB2 (S310F), AKT1 (E17K) and BRAF (V600E) in one patient (4%). See Figures 3D and 4D for the results of OFA analysis in RC. Similarly to what we had observed in patients with CC, few RC patients showed CNAs. In fact, gene amplification of FGFR1 gene (CN=7.5) was observed only in one RC patient, conversely, 5 RC patients showed single SNVs (KRAS, ERBB2, BRAF), 2 RC patients showed the simultaneous presence of mutations in PIK3CA and KRAS, and one RC patient showed mutations in both PIK3CA and AKT1. Again, whereas mutations in BRAF and KRAS were mutually exclusive, RC patients with PIK3CA mutations showed the simultaneous presence of other potentially driver mutations as KRAS and AKT1.

### Absence of the MET (13) – MET (15) fusion in CC and RC

By OFA analysis, we found a high number of CC (n=7) and RC (n=6) that were presumably positive for MET (13) – MET (15) fusion. Importantly, at difference with what observed in LC patients, in the CC/RC samples that were called positive for the MET (13) – MET (15) fusion, the number of reads specific for the fusion was > 20, a value that was higher than limit set by the system to call for the fusion, but much smaller in comparison with the values shown by the fusions reported in the patients LC1 and LC26.

Surprisingly, we were unable to validate the presence of MET exon 14 skipping by RT-PCR in these samples and to identify the genomic alterations that could be considered responsible for the MET(13)–MET(15) fusions in CC and RC, by use of the custom NGS MET panel comprising the genomic region from intron 13 to intron 15.

To confirm that these samples could be considered false positive we diluted RNA from NCI-H596 NSCLC cells that were positive for MET exon 14 skipping, into a RNA extracted from a wild type sample (A549 cells) to determine the limit of detection of OFA (Supplementary Figure 4). Even when the RNA of NCI-H596 cells was diluted 1:1000 (which corresponds to 1 mutant allele out of 2000 cells), the assay detected up to 2200 specific fusion reads.

This led us to conclude that the positive call of the MET(13)–MET(15) fusion in CC and RC was an artefact of the panel, possibly being caused by the low number of specific reads (20) set by the software to call the presence of the fusion.

Another possible explanation for the detection of MET(13)–MET(15) fusion in these samples could be the presence of an incomplete MET transcript identified in ENSEMBL database as ENST00000454623. This transcript includes only the sequence of the exons 13 and 15 of full length transcript and skips exon 14, thus simulating the MET (13)–MET (15) fusion. Although there is no experimental evidence of the ENST00000454623 transcript expression in intestinal cells, it is possible that OFA could detect this isoform, thus releasing an output that includes false positives for the MET(13)–MET(15) fusion.

On the basis of our experiments, we suggest that the threshold in OFA panel to call for the MET (13)–MET (15) fusion should be raised from >20 to >1000 reads.

### Metastatic lymph nodes vs primary tumors

We have also analyzed lymph node metastasis from 3 GC patients (G4-M, G13-M and G20-M) and 3 CC patients (CC29-M, CC32-M and CC33-M). We found that most patients presented the same mutations both in the primary tumors and in the corresponding metastatic lymph nodes, with the exception of patients CC29 and G20, which suggest that metastasis accumulated novel mutations. At difference with the corresponding primary tumors, the metastatic lesion of patient CC29 carries the RAF-1 T421I SNV and the metastatic lesion of patient G20 carries the MET Y1253C SNV. Finally, the CNAs detected in the primary tumors from patients G4 and G13 were also detected in the respective lymph node metastasis, but with a higher copy number with respect to the corresponding primary tumors. See Table 5 for the alterations detected in the lymph node metastasis.

## DISCUSSION

In the present study we have performed NGS analysis by use of OFA to simultaneously detect multiple molecular alterations that included SNVs, CNAs and gene fusions in patients subjected to surgery for LC, GC, CC or RC. Overall, we report that 53% of patients showed at least one potentially druggable alteration. SNVs were identified in 47 patients, CNAs were identified in 10 patients and gene fusions were identified in 3 patients. SNVs were detected in LC, GC, CC and RC; CNAs were consistently more frequent in GC (27%) than in CC or RC (3%); gene fusions were detected only in LC.

The most frequently mutated genes across all tumor types included KRAS, PIK3CA, BRAF, EGFR, NRAS and ERBB2. The genes that were subjected most frequently to CN gains were CCND1, ERBB2, FGFR1 and MYC. Frequently, CN gains co-occurred with other relevant mutations (40% of cases).

LC patients carried activating mutations in EGFR, KRAS, NRAS, PIK3CA or BRAF and the MET(13)–MET(15) or FGFR3-TACC3 fusions. Notably, alterations detected in LC patients involved a single gene, indicating that the identified alterations could drive tumorigenesis in the affected patients. GC patients often carried multiple genetic alterations, with the simultaneous presence of SNVs or CNAs in multiple genes. As a rule, SNVs and CNAs were mutually exclusive in most GC. These results are in agreement with a recent study showing that tumors apparently segregate into two major classes, one primarily dominated by mutations and the other primarily dominated by copy number alterations [35].

The finding that several GC patients carried gain-of-function mutations and/or CN gain of ERBB2 indicated that GC patients may benefit from anti-ERBB2 inhibitors. On the other hand, the presence of the A289D EGFR mutation previously associated with glioma [36], is a novel finding, and suggests potential utility of EGFR inhibitors in gastric cancer.

At difference with what we had observed in GC, CC patients preferentially harboured SNVs. Mutations in BRAF, KRAS and NRAS were mutually exclusive. However, several patients showed the simultaneous presence of SNVs in PIK3CA with other genes. It is worth noting that the A883V RET mutation detected in CC has been previously reported in head and neck cancer [37], suggesting a role for RET activation in the development of CC. Accordingly, we have recently described a gain-of-function MEN2A-like RET mutation (G533C) in a different cohort of CC patients (Oliveira et al., submitted).

Also RC patients harboured preferentially SNVs (KRAS, PIK3CA, ERBB2, AKT1 and BRAF). As in CC patients, mutations in BRAF and KRAS were mutually exclusive, whereas patients with PIK3CA mutations showed the simultaneous presence of other potentially driver mutations.

Overall we have found RAS mutations in 32% of patients, in particular in the KRAS and NRAS isoforms. No mutation in HRAS was identified. In the literature, RAS mutations have been reported to occur in the range of 9–30% of all tumor samples sequenced, with specific RAS isoforms generally differing according to cancer type [38]. In agreement with previous studies [39], we found that the frequency and/or the distribution of RAS mutations were not uniform. In LC patients, RAS mutations were detected in 13.5% of patients, predominantly in the adenocarcinoma histotype. In CC and/or RC patients we found RAS mutations in in 44.5% of cases, a value close to the frequency reported in the literature (52%). KRAS was the predominant mutated isoform. In agreement with previous studies NRAS mutations were found at low frequency in CC or RC [40].

The second most common mutated gene in our cohorts of patients was PIK3CA, which was distributed across all tumor types. Moreover, a gain-of-function mutation of its effector, AKT1, was also identified in CC and RC. However, it is uncertain whether mutant PIK3CA or AKT1 are sufficient oncogenic drivers in this context since they co-existed with additional alterations. Indeed, the coexistence of mutations in KRAS and PIK3CA is frequent in CC and RC patients, and it apparently dictates poor prognosis [41]. On the other hand, while PI3K inhibitorsbuparlisib and alpelisib apparently show activity in preclinical models of multiple solid tumors [42] they have poor efficacy in KRAS-mutant tumors [43].

BRAF was the third most common mutated gene. In fact, the V600E mutation was identified in LC, GC, CC and RC. Expectedly in LC, CC and RC BRAF mutations were mutually exclusive with KRAS mutations [44].

EGFR mutations were identified not only in LC but also in GC, CC and RC. It is of note that the A289D EGFR mutation identified in GC and RC, which represents the most common site of extracellular EGFR mutation, is frequent in human glioma [36]. In agreement with the literature, EGFR mutations in LC patients were detected in former smoker women diagnosed with adenocarcinoma at a frequency of 28% [45].

ERBB2 was mutated in GC and RC and subjected to CN gain in GC and LC. In agreement with previous studies, the tumor type that most frequently showed ERBB2 alteration was GC (25% of cases) [46, 47].

In summary, the results obtained by OFA analysis indicated that on average, 40-70% of patients affected by common cancers harbour molecular alterations that can be potentially targeted by drugs. Two LC patients with EGFR mutant tumors were eligible for treatment with erlotinib and gefitinib, as established by the International Association for the Study of Lung Cancer [48]. On the other hand, at least 5 additional LC patients can be treated with targeted inhibitors such as crizotinib (MET), nintedanib (FGFRs), trastuzumab (ERBB2) or buparlisib (PI3KCA). We have also identified 9 GC, 10 CC and 5 RC patients who could potentially benefit from targeted drugs that include trastuzumab (ERBB2), trametinib (MAP2K1), palbociclib (CDK6), tofacitinib (JAK3) or buparlisib/CH5132799 (PI3KCA) dabrafenib/vemurafenib (BRAF), everolimus (mTOR) or AKT1 inhibitors. Notably, 3 CC patients could be treated with a combination of BRAF and PIK3CA inhibitors.

Patients that presented CN gain of CCND1 could be treated with palbociclib, a highly specific inhibitor of CDK4 and CDK6 [49] whereas patients that presented MYC CN gain could be treated with a synthetic double-stranded RNA that targets the oncogenic MYC protein [50]. On the other hand, CC/RC patients with mutated EGFR, ERBB2, ERBB3 and RET that carry simultaneous activating mutations in KRAS, NRAS and/or BRAF (n=5) are expected to be resistant to tyrosine kinase inhibitors. Conversely, it is to be determined, however, whether PIK3CA- or AKT1-mutated tumors that carry simultaneous mutations in KRAS or NRAS (n=6) are resistant to PIK3CA inhibitors [41].

On the basis of our experience, OFA presents several potential advantages in clinical daily practice including the possibility to detect simultaneously SNVs, CNAs and gene fusions, the requirement of low amount of input nucleic acids, the compatibility with FFPE specimens, the easy detection and interpretation of results. As to the percentage of enrichment in tumor cells, OFA requires standards that are similar to other NGS panels. Time around testing (3 working days with a range of 2–6 days) and costs calculated on a number of consecutive, unselected samples were also comparable or even more convenient in comparison with other methods.

As to the accuracy of mutations detected, all CNAs were validated by another method, and thus they appear completely reliable, since neither false positive nor false negative were detected. In any case, it is to be noted that CNA detection has an intrinsic limitation due to the poor biological comprehension of its consequences in the majority of cases, especially when CN gain is within 2-3 fold.

On the other hand, we found that confirmation of SNVs by Sanger sequencing was possible only when the mutated allele was ≥8% due to poor sensitivity of the technique. Therefore it appears that OFA presents such high sensitivity to allow detection of SNVs and/or small indels even if they are present in rare cellular subpopulations. Conversely, the most important caveat of OFA was the difficulty to confirm gene fusions covered by low number of reads. In fact, we have identified and confirmed the MET(13)–MET(15) fusion in two LC patients that presented a high number of reads (>15000) specific for the fusion transcript but failed to confirm the fusion in CC/RC samples with low number of reads. Notably, a recent study, also using OFA analysis, reported MET(13) – MET(15) fusion in colorectal cancer patients [30]. However, since authors did not exclude that OFA had detected the splice variant of MET, it is unclear how to clinically interpreter their results. The importance of the issue is underlined by a recent study showing the limitation of 8 different amplicon-based DNA NGS panels in the detection of MET(13) – MET(15) fusion [51], in which authors failed to detect the specific MET(13) – MET(15) fusion in almost 40% of cases [51]. Accordingly, the results presented here indicate that the positivity to the MET(13)–MET(15) fusion in CC/RC patients is an artefact of the technique, possibly due to the presence and detection of an alternative mRNA isoform and indicate a potential method to overcome the problems in the identification of MET(13) – MET(15) fusion raised by use of NGS amplicon-based panels.

On the other hand, the inability to confirm the positivity to FGFR3(17)–TACC3(11) fusion may be due to the limited number of cells in which the fusion is present, as demonstrated by the very low number of reads specific for the fusion. On the basis of our experiments as well as published data, we suggest that the threshold in the OFA for the MET(13)–MET(15) fusion call should be raised from >20 to >1000 reads. For this reason we have considered negative all CC/RC patients apparently showing the MET(13)–MET(15) fusion and not informative the NSCLC patient showing the FGFR3(17)–TACC3(11) fusion.

In conclusion, by use of OFA, we have identified several patients that, on the basis of the mutational profiles or their tumors, are potentially eligible for targeted therapy and 11 patients that are potentially resistant to therapy with tyrosine kinase inhibitors, because of simultaneous mutations in KRAS, NRAS and/or BRAF. In a limited number of cases a combined treatment with 2 different inhibitors could be suggested.

## MATERIALS AND METHODS

### Patients

For the set-up of the OFA workflow, FFPE archive slides were retrieved from different cohort of patients subjected to surgical resection for NSCLC (LC, n= 28), gastric (GC, n= 22), colon CC, (n= 31) and rectal (RC, n= 25) cancer. Patients were recruited at AOU Monaldi Hospital (Naples, Italy), IRCCS Fondazione Pascale (Naples, Italy) and/or University Magna Graecia (Catanzaro, Italy). Patients were considered eligible for the study if they had not received any chemotherapy or radiotherapy before they underwent surgery. Metastatic lesions were also retrieved from six patients included in the study. All samples were suitable for molecular analysis. See Supplementary Tables 1-4 for clinical-pathological characteristics of patients.

Median age of patients diagnosed of NSCLC was 69 years (range 57–80) 19 out of 28 were current or ex-smokers. Women were 7 and males were 21. Stage was known for 21 patients: 15 patients had stage IA–IB disease, 5 patients had IIA-IIB and 1 patients had stage III disease. Grade was known for 15 patients: 11 cases were G1-G2, 4 cases were G3.

Additional clinical-pathological information is reported in Supplementary Table 1. As to GC patients, archive material was retrieved from 22 patients. Median age of patients was 70 years (range 51–89). Women were 5 and males were 17. Stage was known for all patients: 5 patients had stage IA–IB disease, 6 patients had IIA-IIB and 4 patients had stage IIIA-IIIB disease and 7 patients and stage IVA-IVB disease. Grade was also known all patients: 9 cases were G1-G2 and 13 cases were G3. Additional clinical-pathological information is reported in Supplementary Table 2.

Archive material was retrieved from 31 CC patients. Median age of patients was 70 years (range 28–86). Women were 14 and males were 19. Grade was known for 29 patients: 21 cases were G1-G2 and 8 cases were G3. Additional clinical-pathological information is reported in Supplementary Table 3.

Archive material was retrieved from 25 RC patients. Median age of patients was 69 years (range 29–81). Women were 8 and males were 17. Grade was known for 19 patients: 11 cases were G1-G2 and 8 cases were G3. Additional clinical-pathological information is reported in Supplementary Table 4.

### Isolation of nucleic acids

For each specimen, 3-10 × 8 μm FFPE sections were microdissected from a single block using scalpel or Laser Capture. Enrichment in tumor cells was evaluted by an expert pathologists (C.M.) and was in the range of 70 to 95%. DNA and RNA were isolated using the Qiagen AllPrep FFPE DNA/RNA Kit (Qiagen, Valencia, CA, USA) following the manufacturer's instructions. Quality and quantity of extracted nucleic acids were assessed using the Qubit Fluorometer (Thermo Fisher Scientific, Waltham, MA, USA) and the 2200 Tape Station instrument (Agilent Technologies, Inc, Santa Clara, CA, USA).

### Library preparation

RNA and genomic DNA were subjected to library preparation prior to sequencing using OFA(Thermo Fisher Scientific). DNA libraries were generated from 10 ng of DNA using the Ion PGM Select Library Kit according to the manufacturer's instructions. RNA libraries were generated from 10 ng of RNA per sample using the Ion PGM Select Library Kit according to the manufacturer's instructions.

### DNA sequencing

OFA allows, in a single workflow that makes use of the Ion PGM Platform, concurrent analysis of DNA and RNA, enabling the identification of single nucleotide hotspot mutations (SNVs) in 35 genes covered by 110 amplicons, copy number alterations (CNAs) in 19 genes covered by 191 amplicons and 23 fusion genes, all in a single workflow using the Ion PGM System. For the different partners involved in the gene fusions detected by OFA see Supplementary Table 5.

Templates for DNA and RNA libraries were prepared using the Ion OneTouch™ Select Template Kit on the Ion One Touch 2 instrument and enriched with the Ion OneTouch ES instrument according to the manufacturer's instructions (Thermo Fisher Scientific).

Sequencing was performed using the Ion PGM™ Select Sequencing Kit on the Ion PGM™ Sequencer (Thermo Fisher Scientific) according to the manufacturer's instructions.

### NGS data analysis

Data analysis was conducted with Torrent Server. Unaligned binary files (uBAM) were uploaded in the Ion Reporter Software (IR) 5.0 (ThermoFisher Scientific) to perform sequence alignment and detection of SNVs, CNVs, 5'-to-3' imbalance and specific gene fusions.

Sequencing data were aligned and mapped to the human hg19 reference genome using the Torrent Suite Server (ver 4.4). Ion Reporter Workflow (version 5.0) was used to identify variants in DNA libraries and fusions in RNA libraries. The automated pipeline of IR 5.0 filtered SNVs for quality, coverage, strand bias and signal shift. Gene annotation was performed using the Oncomine Panel v1.1 Annotations set. SNVs were considered positive if they were covered at least 400 fold, with proportion of reads on target higher than 85%.

Detection of CNAs was performed with IR 5.0 using the Oncomine Panel v2.0 with Baseline and Oncomine Variant annotator v2.0 plugin. CNAs were reported as positive when they presented a MAPD value < 0.4.

For gene fusion detection samples were considered suitable for analysis if at least 40,000 total reads were present and if at least 4 out of 5 expression controls were detected. Tumors were considered positive for a specific fusion if the number of reads reads for a specific fusion was >20. See Supplemental methods for more details.

### Sanger DNA sequencing

Sanger sequencing was performed using BigDye terminator v3.1 (Applied Biosystems, Foster City, CA, USA) with ABI 3100 Genetic Analyzer (Applied Biosystems).

### Quantitative real-time PCR

Results obtained with NGS were validated by quantitative real time PCR (qPCR). GAPDH was used for normalization. Reactions were performed using SYBR Green I PCR Master Mix (Thermofisher Scientific), which included the internal reference (ROX). Each qPCR reaction comprised 10 μl 2× SYBR Green PCR Master Mix, forward and reverse primer at the final concentration of 500 nM.

qPCR reactions were performed using the Quantstudio 12K Flex (Thermo Fisher Scientific). The reaction profile was: initial step, 50°C for 2 min, denaturation, 95°C for 10 min, then 40 cycles of denaturing at 95°C for 15 sec and combined annealing and extension at 60°C for 60 sec. Each qPCR experiment contained triplicates of no-template-controls and patient samples. On the same reaction plate all DNA samples were tested with the test and reference primers.

### Statistical analysis

RT-PCR data are expressed as mean ±SD of at least three independent experiments conducted in triplicates. Statistical significance was evaluated by ANOVA One-way test followed by Dunnett's multiple comparison test. Statistical significance was indicated as follows: p≤ 0.05 (^*^), p≤ 0.01 (^**^), p≤ 0.001 (^***^) and p≤ 0.0001 (^****^).

### Reverse transcriptase PCR (RT-PCR)

We used superscript III retro-transcriptase (ThermoFisher Scentific) to obtain cDNAs and AccuPrime™ Taq DNA Polymerase System (ThermoFisher Scentific) to perform the polymerase chain reaction. For sequences of the primers used, see Supplementary methods.

### Immunohistochemistry

Immunostaining was performed using the avidin-biotin-peroxidase method (LSAB kit; DAKO, Glostrup, Denmark) following standard procedures detailed in [52]. Briefly, FFPE sections were deparaffinized with xylene, rehydrated and microwaved for 5 minutes in 10 mM citrate buffer (pH 6.0). Sections were stained as previously described [53] using anti-HER2 (Signaling Technology Danvers, MA, USA, #4290) and anti-cyclin D1 (Agilent Technologies Santa Clara, CA, #M3642) antibodies. Signal was developed with diaminobenzidine as chromogen. Sections were counterstained with haematoxylin, dehydrated and mounted.

### Fluorescence *in situ* hybridization (FISH)

FISH analysis for HER2 was performed on 5 μm thick slides. Deparaffinization of sections was carried out with two 10 min immersions in bioclear, followed by three 3 min immersions in ethanol 100, 70 and 50%. Briefly, according with manufacturer protocol slides were rinsed in distilled water and immersed in pre-treatment solution at 80°C for 10 min, and in protease solution (previously warmed to 37°C) for 10 min, washed with purified water, air-dried, and dehydrated in ascending grades of alcohol. The commercially available HER2 pharmDX kit (Agilent Technologies Santa Clara, CA, USA, #K5331) was used.

Denaturation and hybridization of the tissue sections were performed using the Thermobrite system (Abbott Molecular Inc. Des Plaines, IL, USA): 75°C for 5 min for the denaturation process and 37°C for 15 hours for the hybridization of the probes. Slides were then washed with 0.4X saline- sodium citrate (SSC) solution at 70°C for 2 min and 2X SSC at room temperature for 3–5 min. Lastly, 10 μL of DAPI was applied on the slides.

Two different investigators that had no previous knowledge of the genetic, clinical and IHC results evaluated FISH analysis.

### Protein extraction and immunoblot

Protein extracts were prepared with lysis buffer containing 50mM HEPES pH 7.5, 5mM EDTA, 250mM NaCl, 1 mM dithiothreitol, 0.5% Nonidet P40, 1mM Na_3_VO_4_, 1mM NaF supplemented with 10μg of aprotinin/ml, 10μg of leupeptin/ml, 1mM PMSF and a mix of protease inhibitors (SIGMA*FAST* protease inhibitor Tablets for general Use). Lysates were centrifuged at 13,000 rpm for 30 min at 4°C and the supernatants were collected. Protein concentration was estimated with a modified Bradford assay (Bio-Rad Laboratories, Berkeley, CA, USA). Western blot analysis was carried out by standard methods and revealed by enhanced chemiluminescence detection using Clarity™ Western ECL Substrate (Bio-Rad Laboratories, Berkeley, CA, USA). The antibody used in MET detection (#8198) was purchased from Signaling Technology (Danvers, MA, USA)

## SUPPLEMENTARY MATERIALS FIGURES AND TABLES




