# Supplementary material for: Simultaneous identification of clinically relevant single nucleotide variants, copy number alterations and gene fusions in solid tumors by targeted next-generation sequencing
Source: Oncotarget. 2018 Apr 27;9(32):22749–68. doi: 10.18632/oncotarget.25229 (PMC5978263; doi:10.18632/oncotarget.25229)
Supplement: Supplementary file 1 [file oncotarget-09-22749-s001.pdf]

Simultaneous identification of clinically relevant single nucleotide variants, copy number alterations and gene fusions in solid tumors by targeted next-generation sequencing

SUPPLEMENTARY MATERIALS

Patients

Median age of patients diagnosed of NSCLC was 69 years (range 57–80) 19 out of 28 were current or ex-smokers. Women were 7 and males were 21. Stage was known for 21 patients: 15 patients had stage IA–IB disease, 5 patients had IIA-IIB and 1 patients had stage III disease. Grade was known for 15 patients: 11 cases were G1-G2, 4 cases were G3.

Additional clinical-pathological information is reported in Supplementary Table 1. As to GC patients, archive material was retrieved from 22 patients. Median age of patients was 70 years (range 51–89). Women were 5 and males were 17. Stage was known for all patients: 5 patients had stage IA–IB disease, 6 patients had IIA-IIB and 4 patients had stage IIIA-IIIB disease and 7 patients and stage IVA-IVB disease. Grade was also known all patients: 9 cases were G1-G2 and 13 cases were G3. Additional clinical-pathological information is reported in Supplementary Table 2.

Archive material was retrieved from 31 CC patients. Median age of patients was 70 years (range 28–86). Women were 14 and males were 19. Grade was known for 29 patients: 21 cases were G1-G2 and 8 cases were G3. Additional clinical-pathological information is reported in Supplementary Table 3.

Archive material was retrieved from 25 RC patients. Median age of patients was 69 years (range 29–81). Women were 8 and males were 17. Grade was known for 19 patients: 11 cases were G1-G2 and 8 cases were G3. Additional clinical-pathological information is reported in Supplementary Table 4.

DNA sequencing

The Oncomine™ Focus Assay (Thermofisher.com) is a next-generation sequencing panel that includes 52 genes associated with solid tumors that can be targeted by drugs.

Genes analysed for the presence of SNVs include: AKT1, ALK, AR, BRAF, Cdk4, CTNNB1, DDR2, EGFR, ERBB2, ERBB3, ERBB4, ESR1, FGFR2, FGFR3, GNA11, GNAQ, HRAS, IDH1, IDH2, JAK1, JAK2, JAK3, KIT, KRAS, MAP2K1, MAP2K2, MET, MTOR, NRAS, PDGFRA, PIK3CA, RAF1, RET, ROS1, SMO.

Gene potentially subjected to CNAs include: ALK, AR, BRAF, CCND1, Cdk4, Cdk6, EGFR, ERBB2, FGFR1, FGFR2, FGFR3, FGFR4, KIT, KRAS, MET, MYC, MYCN, PDGFRA, PIK3CA.

Genes candidate for fusions include: ALK, RET, ROS1, NTRK1, NTRK2, NTRK3, FGFR1, FGFR2, FGFR3, MET, BRAF, RAF1, ERG, ETV1, ETV4, ETV5, ABL1, AKT3, AXL, EGFR, ERBB2, PDGFRA, PPARG.

NGS data analysis

For the identification of SNVs and small indels, the IR 5.0 integrated the Torrent Variant Caller (TVC) algorithm optimized for sequencing data obtained on the Ion Torrent platform. The automated pipeline of IR 5.0 filters SNVs for quality, coverage, strand bias and signal shift. All SNVs called as positive were covered at least 400X.

The CNV calling Algorithm in IR 5.0 makes use of the Median of the Absolute values of all Pairwise Differences (MAPD) between log2 ratios per tile for a given run to give a measurement of noise and/or other errors. The observed difference is due to CNVs (all CNV reported presented a MAPD value < 0.4).

The fusion analysis pipeline processes 3 input files (an uBAM file, a fusion reference Fasta file comprising chimeric sequences for targeted fusion transcripts and control targets, a target bed file that includes information about fusion breakpoint, expression controls and target assays. The algorithm processes the input files in order to extract read counts for fusion and expression controls. Parameters set to obtain accurate imbalance determinations were: a threshold of 40,000 total reads/sample, presence of ≥4 out of 5 expression controls and >20 reads for a specific fusion.

Primers used for fusion validation

The primers for RT-PCR in validation of MET exon 14 skipping were as follows:

FW, 5′- CTT CAA CCG TCC TTG GAA AA -3′;

REV, 5′- CCTATGACTTCATTGAAATGCAC -3′.

The primers used for RT-PCR in validation of FGFR3-TACC3 fusion were as follows:

FW: 5’- CGTGAAGATGCTGAAAGACGATG – 3’

REV: 5’ – AAACGCTTGAAGAGGTCGGAG – 3’

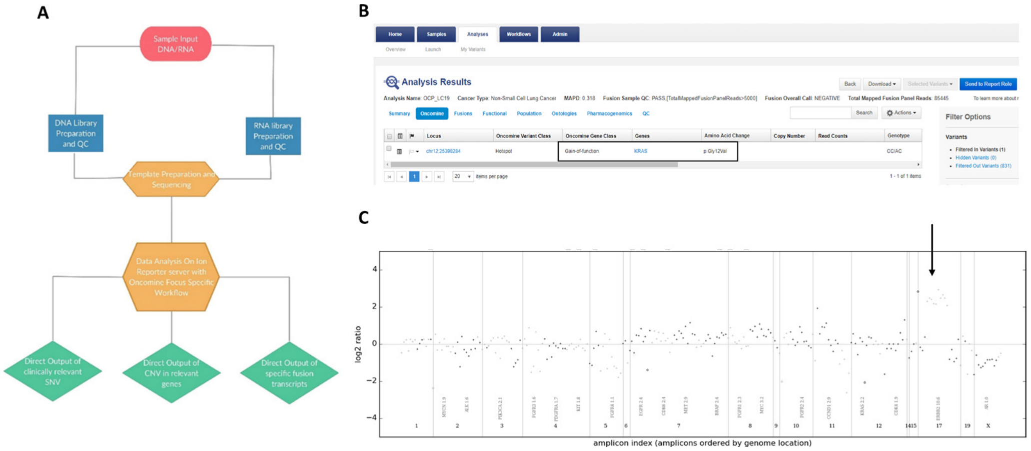

Supplementary Figure 1: Oncomine™ Focus and representative output. (A) FFPE material was used to prepare RNA/DNA libraries, which were loaded onto an Ion Select 318 chip and run onto the Ion PGM machine. Automated analysis of data for detection of SNV, CNV, and gene fusions was performed with the Ion Reporter Workflow Software. **(B)** Representative output of the Oncomine™ Focus – Ion Reporter Variant Detection, with the indication of the gene, nucleotide variant, type of alteration and the targeting drug. **(C)** Representative output of the Oncomine™ Focus – Ion Reporter CNA Detection, with the indication of the gene, nucleotide variant, type of alteration and the targeting drug.

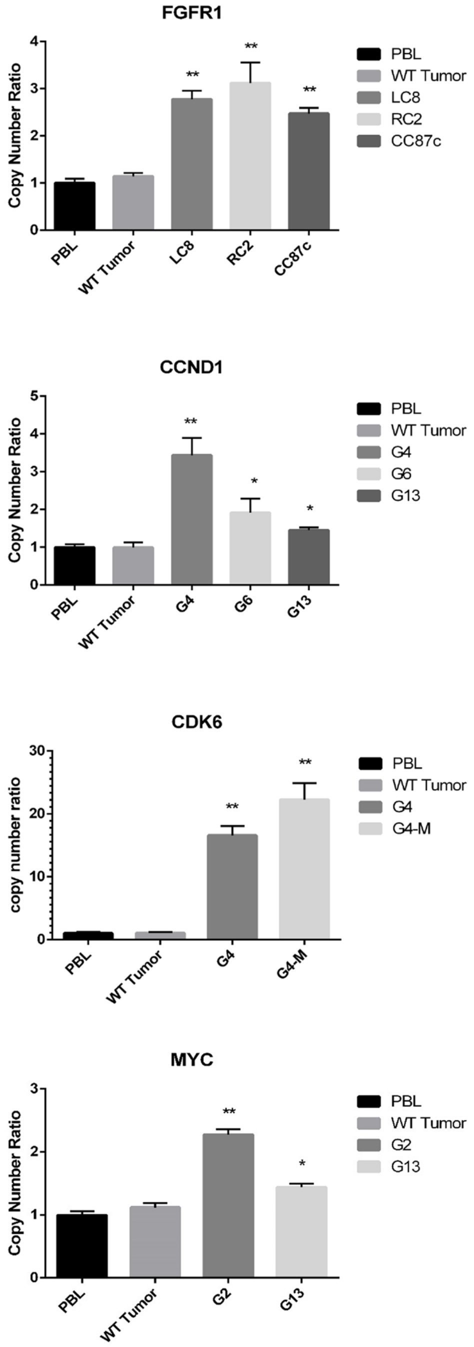

Supplementary Figure 2: Validation of Copy number amplifications detected by Oncomine™ Focus Assay. Quantitative realtime PCR inpatients with amplified genes encoding FGFR1 (patients CC4, LC8, RC2), CDK6 (G4, G4-M), CCND1 (G6, G13, G4) and MYC (patients G2, G13). PBL, peripheral blood lymphocytes; tumors showing noamplification of FGFR1, CDK6 or CCND1, respectively. Statistical significance indicated bynumber of stars in each patient when confronted with PBL.

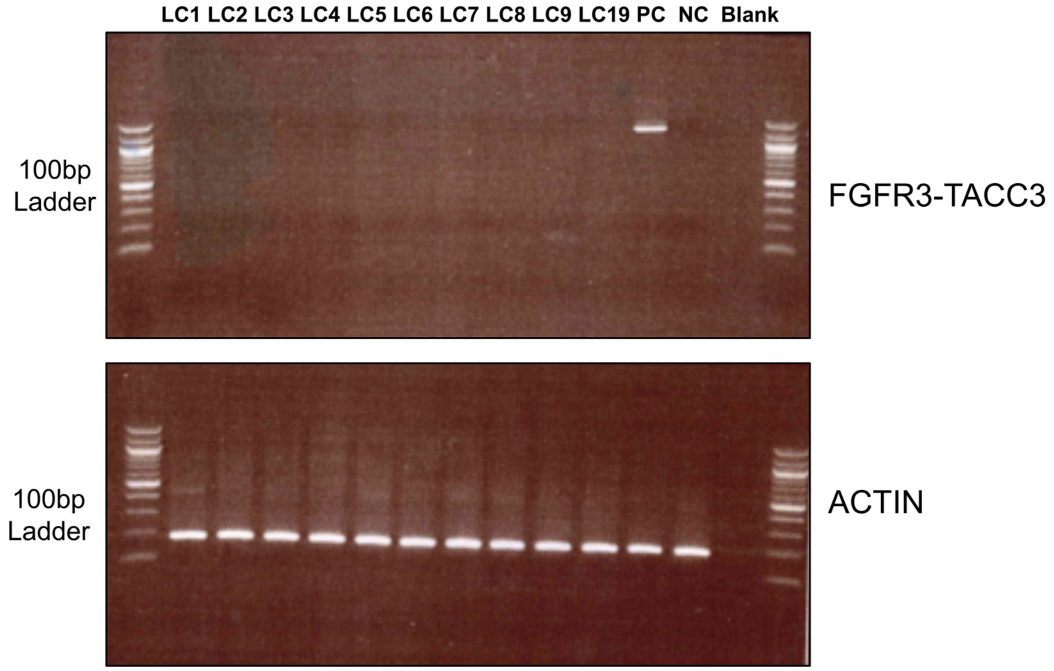

Supplementary Figure 3: Analysis of FGFR3-TACC3 fusion by semi-quantitative RT-PCR in LC Patients. Representative RT-PCR analysis for detection of FGFR3-TACC3 fusion in LC samples included in the study. A positive control (PC), negative control (NC) and blank (Blank) were included. Actin was used as internal control of the RNA used.

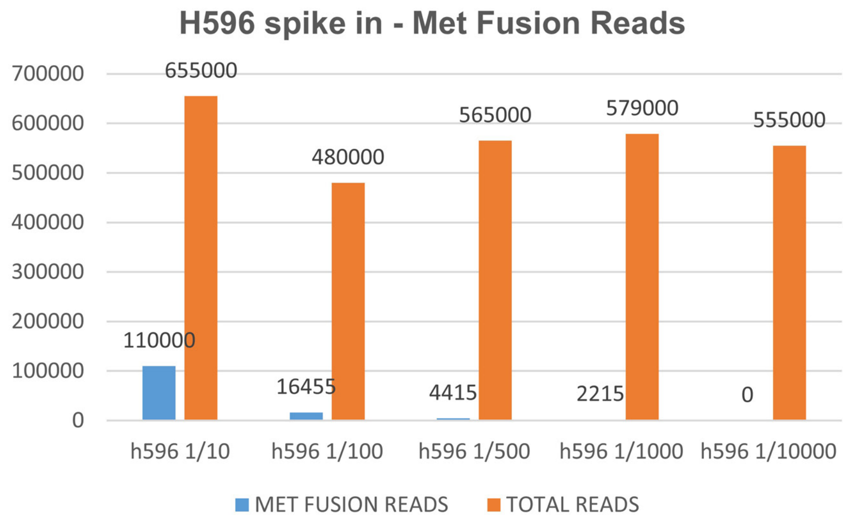

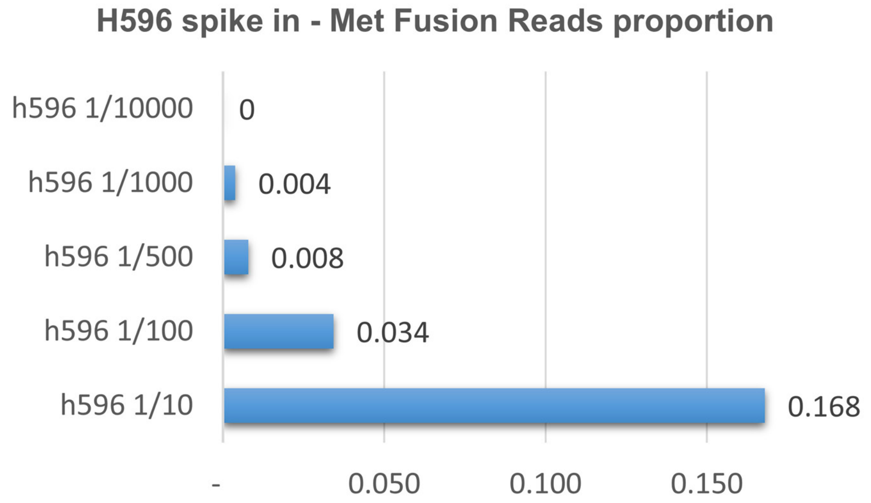

Supplementary Figure 4: Determination of OFA sensitivity in identification of specific MET fusions. (A) Limiting dilution (from 1:10 to1:10,000) of RNA from the MET-positive NCI-H596 cells into the RNA from the MET fusion negative A549 cells. **(B)** Oncomine™ Focus Assay of RNA from NCI-H596 cells diluted (from 1:10 to 1:10,000) into the RNA from MET fusion-negative A549 cells. Blue bar, MET fusion specific reads; brown bar, total reads.

Supplementary Table 1: Clinical-pathological characteristics of LC patients included in the study

Patient	Gender	Age (years)	Smoking Status	WHO	Grade	Stage
LC1	M	68		Adenosquamous Carcinoma		
LC2	M	74	smoker	Adenocarcinoma	G2	IA
LC3	F	69	no smoker	Other		
LC4	M	75	ex smoker	Other	G2	IB
LC5	M	67	ex smoker;	Squamous cell carcinoma	G2	IIA
LC6	M	75	ex smoker	Bronchioloalveolar carcinoma	G2	IB
LC7	M	66	smoker	Adenocarcinoma	G2	IB
LC8	M	80		Squamous cell carcinoma	G2	IB
LC9	M	62	smoker	Squamous cell carcinoma	G2	IB
LC10	M	68	smoker	Squamous cell carcinoma	G3	IIB
LC11	M	69	smoker	Large Cell Carcinoma	G3	IB
LC12	F	76	no smoker	Adenocarcinoma	G2	IB
LC13	M	65	ex smoker	Adenocarcinoma	G2	IB
LC14	F	66	ex smoker;	Bronchioloalveolar carcinoma		
LC15	M	57	ex smoker	Other	G2	IB
LC16	M	44	ex smoker	Adenocarcinoma	G2	IIB
LC17	M	75	smoker	Bronchioloalveolar carcinoma	G2	IB
LC18	M	54	ex smoker	Large Cell Carcinoma	G3	IIB
LC19	M	74	ex smoker	Squamous cell carcinoma	G2	IIB
LC20	M	63	smoker	Adenocarcinoma	G2	IB
LC21	F	77	ex smoker	Squamous cell carcinoma		
LC22	M	59	ex smoker	Squamous cell carcinoma	G2	IB
LC23	F	70	ex smoker	Squamous cell carcinoma	G2	IB
LC24	F	65		Adenocarcinoma	G2	IB
LC25	F	71	ex smoker	Adenocarcinoma	G2	IB
LC26	M	75	no smoker	Squamous cell carcinoma	G3	IIIA
LC27	M	73	ex smoker	Squamous cell carcinoma	G3	IIB
LC28	M	70	no smoker	Adenocarcinoma		

Supplementary Table 2: Clinical-pathological characteristics of GC patients included in the study

Patient	Gender	Age (years)	T	N	M	Grade	WHO	Stage
G1	M	77	T3	N1	M1	G2	Intestinal	IV
G2	F	51	T4	N1	M0	G3	Signet ring cell carcinoma	IV
G3	F	70	T3	N2	M0	G3	Signet ring cell carcinoma	IIIB
G4	M	69	T2	N1b	M1	G2	Tubulo-papillary gastric adenocarcinoma	II
G5	M	57	T4a	N3a	M0	G2	Signet ring cell carcinoma	IV
G6	M	79	T2b	N1	M0	G2	Signet ring cell carcinoma	II
G7	M	77	T3	N2	M0	G3	Intestinal	III
G8	M	78	T2b	N2	M0	G3	Poorly Differentiated	III
G9	F	73	T2	N1	M0	G3	Intestinal	II
G10	F	89	T3	N0	M0	G2	Intestinal	II
G11	M	69	T3	N1	M0	G3	Intestinal	IIIA
G12	M	70	T2b	N0	M0	G2	Intestinal	I
G13	M	58	T3	N3	M1	G3	Signet ring cell carcinoma	IV
G14	M	81	T1b	N1	M0	G2	Intestinal	IB
G15	M	48	T1	N0	M0	G1	Intestinal	I
G16	M	77	T2a	N0	M0	G2	Intestinal	I
G17	F	72	T1	N0	M0	G3	Diffuse	I
G18	M	75	T3	N3	M0	G3	Poorly Differentiated	IV
G19	M	54	T2	N1	M0	G3	Intestinal	II
G20	M	72	T2	N3	M1	G3	Signet ring cell carcinoma	IV
G21	M	64	T3	N3	M1	G3	Poorly Differentiated	IV
G22	M	82	T3	N0	M0	G3	Intestinal	II

Supplementary Table 3: Clinical-pathological characteristics of CC patients included in the study

Patients	Gender	Age (years)	Segment	T	N	M	Grade
CC1	M	58	Transverse Colon	T3	N0	M0	G2
CC2	F	50	Descending Colon				
CC3	M		Transverse Colon	T3	N1a	M0	G2
CC4	M	79	Cecum	T3	N0	MX	G2
CC5	M	48	Descending Colon	T2	N0	M0	G1
CC6	M	65	Transverse Colon	T3	N2b	Mx	G2
CC7	F	78	Cecum	T2	N0	Mx	G2
CC8	F	70	Hepatic Flexure	T4a	N2b	MX	G3
CC9	M	68	Descending Colon	T4a	N1b	MX	G2
CC10	M	78		T3	N0	Mx	G2
CC11	M	75		T3	N0	Mx	G2
CC12	M	59	Ascending Colon	T3	N2b	Mx	G2
CC13	M	75	Descending Colon	T3	N0	M0	G2
CC14	M	67	Descending Colon	T3	N2	M0	G3
CC15	F	84	Hepatic Flexure				
CC16	M	75	Ascending Colon	T4a	N2a	MX	G2
CC17	F	84	Splenic Flexure	T3	N1	M0	G2
CC18	M	79	Descending Colon	T3	N0	M0	G2
CC19	M	56	Descending Colon	T4	N1a	M0	G3
CC20	F	86	Ascending Colon	T3	N0	M0	G2
CC21	M	71	Descending Colon	T3	N0	M0	G2
CC22	F	67	Descending Colon	T3	N1B	MX	G2
CC23	M	68	Descending Colon	T3	N0	M0	G3
CC24	M	56	Descending Colon	T2	N0	MX	G2
CC25	F	56	Descending Colon	T4	N0	M0	G3
CC26	F	46	Descending Colon	T4	N1b	M0	G3
CC27	F	89	Descending Colon	T3	N1b	M0	G3
CC28	M		Ascending Colon	T3	N1b	M1	G2
CC29	F	84	Descending Colon	T4b	N0	M1	G2
CC30	F	85		T4a	N1b	M1	G2
CC31	F	70	Descending Colon	T4	N2a	M1	G3

Supplementary Table 4: Clinical-pathological characteristics of RC patients included in the study

Patient	Gender	Age (years)	T	N	M	Grade
RC1	M	72	T3	N1B	Mx	G3
RC2	M	29	T4a	N2b	M0	G3
RC3	M	76	T2	N0	M0	G2
RC4	M	78	T2	N0	MX	G2
RC5						
RC6	M	64		N0	M0	
RC7	M	78	T3	N0	MX	G2
RC8	M	79	T2	N0	M0	G2
RC9	F	63	T4	N1	M0	G3
RC10	M	76		N0	M0	
RC11	F	61	T4a	N1	MX	G3
RC12	M	78	T3	N0	MX	G2
RC13	M	80	T2	N1	M0	G2
RC14	F	71	T2	N0	MX	G3
RC15	M	73				G3
RC16	M	63	T3	N1	MX	G2
RC17	M	73	T4a	N2b	MX	G2
RC18	M	81	T3	N0	M0	G3
RC19	F	81	T2	N0	Mx	G2
RC20	M	59	T4b	N1	M0	G3
RC21	F	69	T2	N0	Mx	G2
RC22	F	60	NA	NA	NA	NA
RC23						
RC24	F					

Supplementary Table 5: List of gene fusions detected by the oncomine focus assay

See Supplementary File 1
